# Association between spinal manipulative therapy and cervical artery dissection in patients with neck pain or headache: a retrospective cohort study

**DOI:** 10.1186/s12998-026-00678-4

**Published:** 2026-09-14

**Authors:** Robert J. Trager, Anthony N. Baumann, Collin M. Labak, Kelsey L. Lewis, Jaime Abraham Perez, Christine M. Goertz, Andres Schuster

**Affiliations:** 1 Connor Whole Health, University Hospitals Cleveland Medical Center, Cleveland, OH USA; 2 Department of Family Medicine and Community Health, Case Western Reserve University School of Medicine, Cleveland, OH USA; 3 Department of Biostatistics and Bioinformatics Clinical Research Training Program, Duke University School of Medicine, Durham, NC USA; 4 Department of Orthopaedic Surgery, Cleveland Clinic Akron General, Akron, OH USA; 5 Department of Rehabilitation Services, University Hospitals, Cleveland, OH USA; 6 Department of Neurosurgery, University Hospitals Cleveland Medical Center, Cleveland, OH USA; 7 Clinical Research Center, University Hospitals Cleveland Medical Center, Cleveland, OH USA; 8 Duke Clinical Research Institute, Duke University School of Medicine, Durham, NC USA; 9 Duke-Margolis Institute for Health Policy, Duke University, Durham, NC USA; 10 Harrington Heart and Vascular Institute, University Hospitals Cleveland Medical Center, Cleveland, USA

**Keywords:** Chiropractic, Spinal manipulation, Adverse outcome pathways, Safety, Vertebral artery dissection

## Abstract

**Objectives:**

Cervical artery dissection (CeAD) has been proposed as a sequela of spinal manipulative therapy (SMT), however, protopathic bias arising from care-seeking for CeAD symptoms (e.g., neck pain/headache) may overestimate observed associations. Among adults experiencing new neck pain/headache, we tested the null hypothesis of no significant CeAD risk within 30 days following SMT compared to matched ibuprofen-prescribed controls.

**Methods:**

We searched United States TriNetX Linked health records/claims data (2010–2025) for adults seeking care for new episodes of neck pain/headache receiving (1) chiropractor-administered cervical SMT or (2) outpatient visit with ibuprofen prescription, excluding those with previous cerebrovascular disease and/or CeAD, using propensity matching to minimize confounding. We examined the risk ratio (RR) of CeAD (primary outcome), exploring cumulative incidence, CeAD subtypes, absolute risk difference (RD), and negative control outcomes.

**Results:**

After matching, there were 431,492 patients per cohort. In the SMT cohort, compared with the ibuprofen cohort, CeAD risk was lower while RD was minimal [95% CI] (RR = 0.53 [0.35, 0.83]; RD=-6.3 [-10.8, -2.0] events per 100,000 patients; *P* = 0.004). Carotid artery dissection risk was lower; vertebral artery dissection risk was not significantly different. Data quality supported balanced confounders and minimal follow-up loss (< 3%). Negative control outcomes were partially balanced.

**Conclusions:**

This study found no evidence of an increased risk of CeAD after chiropractor-administered cervical SMT compared with ibuprofen prescription in adults seeking care for new episodes of neck pain or headache. Despite the rarity of these events, all clinicians, including chiropractors, should maintain vigilance for CeAD symptoms. Additional multidisciplinary research is warranted to further address potential confounding and examine patient subgroups (e.g., migraine) and stroke.

**Supplementary Information:**

The online version contains supplementary material available at https://doi.org/10.1186/s12998-026-00678-4.

## Introduction

Cervical artery dissection (CeAD) is characterized by a tear in the intimal layer of the vertebral or carotid arterial wall and development of associated intraluminal hematoma and subsequent thrombus, and may result in embolic stroke [1]. In the general population, the incidence of CeAD has been estimated at 4.7 (95% CI, 3.9–5.5) per 100,000 person-years between 2002 and 2020, with rates increasing from 2.3 (95% CI: 1.1–3.5) per 100,000 person-years during 2002–2006 to 8.9 (95% CI: 6.5–11.3) during 2017–2020 [2]. Initial case series suggested that CeAD may rarely occur soon after spinal manipulative therapy (SMT) [3], a treatment commonly used by chiropractors and other clinicians to treat neck pain and headache [4]. While a recent meta-analysis found no significant association between SMT and CeAD, the compiled evidence was very low certainty, being hindered by risk of bias, inconsistency, indirectness, and imprecision [5].

Some researchers have proposed that the thrust and/or positioning used in cervical SMT may injure the cervical arteries [6]. However, supporting mechanistic evidence is limited. One cadaver-based study found that the vertebral artery elongates without stretch during SMT [7]. Other studies found no changes in carotid or vertebral arterial blood flow following cervical SMT in healthy volunteers or individuals with chronic nonspecific neck pain [8–10]. However, evidence from routine clinical populations is needed, considering patients may have comorbidities or pre-existing but undiagnosed CeAD not represented in these studies.

Although certain case-control and case-crossover studies have found a positive association between SMT and CeAD [11–14], these findings may be explained by patients seeking SMT for neck pain or headache resulting from a pre-existing CeAD. These methodological biases have been described as protopathic bias, confounding by indication, and reverse causation [13, 15–17]. Neck pain and headache are common reasons for seeking chiropractic care [4], and also common symptoms of CeAD [18, 19].

Interpretation of previous research is further complicated by methodological considerations. Certain case-control studies which identified a positive association between SMT and CeAD evaluated SMT as one among many potential risk factors rather than as the primary exposure of interest [11, 20, 21]. This may increase type I error, reduce events-per-variable, and necessitate inclusion of a broader, more heterogeneous sample. Further, these studies used hospitalized stroke patients as comparators, who typically had a higher prevalence of traditional vascular comorbidities such as hypertension, and therefore may have been less likely to receive SMT, potentially augmenting the observed SMT-CeAD risk association [22–24]. This comparator choice is recognized to influence CeAD associations [5], which is potentially explained by the divergent risk factors between CeAD and stroke [25].

While numerous studies have investigated vascular events following SMT, prior research has not uniformly isolated CeAD as an independent outcome. Multiple studies used composite outcomes of CeAD, arterial occlusion or injury, and stroke [17, 20, 26–28], hindering risk assessment for CeAD versus other vascular events [15, 29]. Only 56% of CeADs result in stroke [30]. CeAD has distinct risk factors compared to stroke [31], being more associated with younger age, migraines, minor trauma, and less with traditional atherosclerotic or cardiovascular risk factors [11, 21, 31]. Given the limited certainty regarding SMT and CeAD, additional robust studies are needed [5, 15].

This retrospective cohort study examined the association between SMT and CeAD in adults treated for new neck pain and/or headache over a one-month follow-up. We tested the null hypothesis of no significant association between SMT and CeAD compared to matched controls receiving ibuprofen, a nonsteroidal anti-inflammatory drug (NSAID) commonly prescribed for neck pain and/or headache. Secondarily, we explored cumulative incidence of CeAD and risk of CeAD subtypes.

## Methods

### Study design

This study adhered to a registered protocol [32]. Analytic additions beyond the protocol included reporting of absolute risk differences, E-values, hazard ratios, and a description of between-cohort crossover during follow-up, each included to aid interpretation or address peer reviewer comments. We included patients meeting eligibility criteria spanning 2010 to 2025, 15 years to one month prior to the query date of September 11, 2025, in a longitudinal retrospective analysis. Inpatient and outpatient data were sourced from the United States TriNetX Linked Network (TriNetX, LLC, Cambridge, MA, US), a resource linking electronic health records with medical claims, pharmacy claims, cancer registries, and mortality data [33–35], with 19,679,930 unique patients. Data are de-identified and compliant with the Health Insurance Portability and Accountability Act. The network includes closed claims from over 100 US payers, including commercial insurers, Medicare Advantage, and Medicaid, and medical records from 22 large and/or academic US healthcare organizations. Internal validation studies have demonstrated 99% precision in patient linkage across datasets [33, 36]. Clinical and billing data include demographics, diagnoses, procedures, medications, and lab results, and are coded using International Classification of Diseases, 10th Revision (ICD-10), Current Procedural Terminology (CPT), Veterans Affairs National Drug File (VA), and other nomenclatures. ICD-10 codes are automatically interconverted to ICD 9th Revision codes by TriNetX for older records as needed, using General Equivalence Mappings. Researchers can query cohorts and analyze outcomes within the online TriNetX platform. TriNetX Linked does not transform dates of service or use date-shifting. Patients with gaps in insurance coverage remain represented in the dataset. Although comparative data completeness metrics are unavailable, linked health records and claims generally have better event capture than either source alone [37]. 

The University Hospitals Institutional Review Board (IRB; Cleveland, OH, US) considers studies using de-identified data from the online TriNetX platform ‘Not Human Subjects Research’ thereby exempting the present study from IRB review and waiving the need for consent.

### Participants

#### Eligibility criteria

We included adults at least age 18 years with a new episode of neck pain or headache, divided into two cohorts based on initial treatment. Each eligible patient was included only once, with the first qualifying index event defining cohort assignment. The SMT cohort included patients treated with chiropractor-administered SMT (CPT: 98940, 98941, 98942) plus a diagnosis indicating segmental dysfunction of the cervical spine (ICD-10: M99.01), aiming to capture cervical SMT based on typical procedure/diagnosis pairing. The control cohort was patients treated with ibuprofen prescription (RxNorm: 5640). Neck pain and headache diagnoses included symptom-based codes, and common or prevalent neck pain and headache conditions [38, 39] (Supplemental Table S1). We focused on SMT administered by chiropractors given the specificity of its CPT codes [40], exclusivity to treatment of the spine, and adequate sample size. We avoided codes describing osteopathic manipulation and manual therapy techniques, which are not necessarily specific to the spine or representative of joint manipulation [41, 42]. Further, the patient populations and techniques associated with osteopathic care may differ in ways that would require separate study design considerations.

We required individuals receiving ibuprofen prescription to be seen in an outpatient setting (CPT: 1013626 or visit: ambulatory) on the same date as the prescription. This aimed to ensure comparable patient complexity considering chiropractors practice in outpatient settings. NSAIDs are one of most commonly prescribed medications for neck pain [43] and headache [44]. Ibuprofen is a commonly used NSAID, inexpensive, and often prescribed in ambulatory settings, thereby making it a useful active comparator. Although prolonged or high-dose NSAID use, including ibuprofen, has been associated with increased cardiovascular thrombotic risk, including ischemic stroke [45], these outcomes are mechanistically distinct from CeAD. NSAID or ibuprofen exposure has not been identified as an established risk factor for CeAD in contemporary reviews [5, 46], and studies have found no significant association between ibuprofen and cerebrovascular events up to four weeks after prescription [47, 48]. Ibuprofen prescription was therefore considered a clinically relevant active comparator for the exposure of interest (i.e., SMT), although prior or over-the-counter use, medication non-adherence, and an unrecognized CeAD-specific association could not be definitively excluded. Both ibuprofen and cervical SMT are not indicated for treatment of acute cerebrovascular events [16, 49–52]. Accordingly, our design aimed to standardize initial encounters to those where clinicians did not suspect acute cerebrovascular events.

We required patients to have a preceding healthcare visit of any kind between one day and two years prior to the index visit, to improve data completeness. We excluded individuals with any history of cerebrovascular disease (e.g., stroke and related conditions), CeAD or cervical artery surgery, serious spinal pathology, and those with emergency care in the preceding two weeks, considering CeAD may be missed among individuals with neck pain or headache [53]. We also excluded those with recent anterior neck surgery, spine surgery, critical care, or inpatient care (i.e., in the preceding two weeks) [18]. To help define new episodes of care for neck pain/headache, we excluded patients with any of the eligible neck pain or headache diagnosis codes in the 30 days preceding the index date [54]. Eligibility criteria and the corresponding time windows for exclusions are summarized in Supplemental Figure S1 and Table S2.

### Variables

We implemented propensity score matching to balance confounding variables with known associations with CeAD including demographics, comorbidities, medications, and inpatient and emergency visits and cardiology procedures to account for patient complexity (Figure S2; Table S3), using a continuous one-year lookback period preceding the index date for covariate data. Accordingly, covariates were eligible if recorded at any point during the 365 days immediately prior to the index date, while age (specifically “age at index”) was determined on the index date itself. To minimize time-related bias from increasing incidence/diagnosis of CeAD [2], we matched patients by age at index date and current age, to balance age distributions and years of exposure.

### Primary outcome

We ascertained a composite of diagnoses of CeAD (ICD-10: I77.71 [carotid] or I77.74 [vertebral]) over a 30-day follow-up window commencing at the index date. The positive predictive value of ICD coding for CeAD is 90% [55]. A specific outcome isolating CeAD, rather than stroke or composite cerebrovascular outcomes, was selected because CeAD represents the underlying vascular pathology of interest, whereas ischemic stroke is a downstream complication that occurs in only a subset of patients with CeAD [30]. Focusing on CeAD also aligned with our prespecified study objectives, avoided multiplicity associated with evaluating multiple outcomes, and allowed eligibility criteria and confounder adjustment to focus on established CeAD-specific risk factors, which differ in part from those for stroke [11, 21, 25, 31].

Previous studies reported symptoms of CeAD after cervical SMT began within one week, but patients delayed up to two weeks before seeking care [14, 56–58]. Other studies reported significant associations between minor mechanical events affecting the head and neck one month prior to CeAD, yet not over the day or week prior [59, 60], suggesting a longer window was needed. CeAD diagnosis is often delayed (mean 9 days; standard deviation [SD] = 12) [61], with delays up to four weeks [62–64]. Finally, with real-world data, shorter windows may miss relevant CeADs due to miscoding or billing lags [65].

This study avoided examining stroke as an outcome, which would require different eligibility criteria and propensity matching variables [31].

### Secondary outcomes

We plotted cumulative CeAD incidence over 30 days’ follow-up to provide insight into timing of events and explored incidence and risk of CeAD subtypes (carotid and vertebral artery dissection).

To further evaluate matching success and unmeasured confounding, we examined negative control outcomes unrelated to SMT or CeAD [66–68], including colonoscopy, magnetic resonance imaging (MRI) of the lower extremity, foreign body removal, and appendectomy, considering risk ratios (RRs) from 0.73 to 1.38 balanced [69]. Negative control outcomes were selected during protocol development based on: (1) content knowledge and/or established absence of a plausible mechanism linking them to SMT or CeAD, and (2) requirement of a similar confounding structure (i.e., general healthcare engagement and acute care-seeking behavior) with the outcome of interest of CeAD [66]. We prioritized procedures over diagnosis codes where possible to better ensure true healthcare activity, given the absence of a diagnostic washout period for these outcomes. Additional candidate outcomes such as medications, vaccine administration, or imaging procedures were considered but excluded due to anticipated low event counts, overlap with cerebrovascular diagnostic workup, and a hard limit of outcomes allowed by TriNetX software.

We explored mean counts of SMT visits and ibuprofen prescriptions, and receipt of physical therapy evaluation (CPT: 1029677; encompassing initial evaluations of low, moderate, and high complexity and re-evaluations [97161–97164]) as a marker of related musculoskeletal care-seeking during follow-up to further assess cohort comparability. Study outcomes are detailed in Supplemental Table S4.

### Statistical methods

Propensity matching was conducted using the online TriNetX platform, with 1:1 greedy nearest-neighbor matching and caliper of 0.1 pooled standard deviations. We assessed covariate balance using standardized mean difference (SMD) using a threshold of > 0.1 for imbalance [70]. We reported mean and median duration of follow-up in days, and proportion of patients having complete follow-up. The primary outcome was RR for CeAD, with RRs calculated from contingency tables. We added absolute risk difference to align with sample size calculation and facilitate interpretation, using the Miettinen and Nurminen method via DescTools [71], and E-value analysis to evaluate unmeasured confounding (Table S5). Chi-square tests were used for P-values. We plotted propensity score density, covariate balance, and total and cumulative incidences with 95% confidence intervals (CI) using R (version 4.2.2, Vienna, AT [72]) and ggplot2 [73]. As the TriNetX platform returns aggregate rather than patient-level data, formal imputation of missing values was not feasible. Data completeness was addressed through eligibility criteria, and described through demographics and other data quality assessments.

Follow-up care metrics were explored over the same 30-day window as largely pre-specified data quality indicators, later bolstered by crossover metrics, to overall provide insight into exposure dose, care-seeking patterns, and between-cohort comparability, and were interpreted using contemporary real-world observational literature and clinical practice guideline recommendations.

As a post hoc analysis, time-to-event outcomes were explored in the propensity score-matched cohorts using Kaplan-Meier methods and Cox proportional hazards modeling within the TriNetX platform (R Survival package version 3.2-3). Hazard ratios (HRs) with 95% confidence intervals were estimated, and the proportional hazards assumption was evaluated using Schoenfeld residual-based tests, with *P* > 0.05 interpreted as consistent with proportional hazards. For time-to-event analyses, patients contributed available clinical data for up to 30 days after the index event. Patients were censored the day after their last recorded clinical fact if it occurred within the 30-day follow-up window. Mortality was not treated as a censoring event.

We estimated that a total sample size of 676,160 patients (338,080 per cohort) would be required using data from a previous study [27]. Calculations used G*Power (Kiel University, Germany) z-tests to detect a difference in incidence proportions between cohorts of 0.0003 (0.0016 vs. 0.0013) for CeAD using a power of 0.90, 1:1 allocation, and two-tailed alpha of 0.05.

Our study design allowed for natural treatment crossover (contamination) during the follow-up in both cohorts, with crossover from the ibuprofen to SMT cohort permitted after 7 days following the index date. This choice aimed to maximize sample size, reflect real-world clinical practice, and enhance generalizability, and the extent of crossover was described along with any potential limitations.

## Results

### Participants

Before matching, there were 489,838 patients in the SMT cohort and 919,171 in the ibuprofen cohort (Figure S3). Cohorts were initially imbalanced (SMDs > 0.1 for select variables; Table 1). For example, the SMT cohort had lower proportions of patients who identified as Black or African American, or had emergency or inpatient visits, or substance use disorder. After matching, both cohorts contained 431,492 patients and were balanced (SMDs < 0.1) with similar ages (SD) (SMT: 43.4 [14.1]; ibuprofen 43.5 [14.6]), and female predominance (SMT: 65%; ibuprofen: 66%).

**Table 1:** Baseline characteristics before and after propensity score matching

Variable *n* (%) or mean (SD)	Before matching			After matching		
	SMT	Ibuprofen	SMD	SMT	Ibuprofen	SMD
N	489,838	919,171	NA	431,492	431,492	NA
Age at index	43.4 (14.1)	42.3 (14.6)	0.078	43.4 (14.1)	43.5 (14.6)	0.007
Current age	49.8 (14.7)	49.3 (14.7)	0.034	50.0 (14.7)	50.0 (14.8)	0.001
Female	307,369 (63%)	653,059 (71%)	0.177	281,533 (65%)	283,470 (66%)	0.009
Substance use disorder	43,576 (9%)	188,341 (20%)	0.332	43,279 (10%)	43,293 (10%)	0.000
Ehlers-Danlos syndromes	737 (0%)	818 (0%)	0.018	576 (0%)	574 (0%)	0.000
Syncope	9152 (2%)	26,604 (3%)	0.067	8650 (2%)	8665 (2%)	0.000
Asian	6785 (1%)	17,298 (2%)	0.039	6762 (2%)	6779 (2%)	0.000
Other race	34,510 (7%)	92,908 (10%)	0.110	33,877 (8%)	33,942 (8%)	0.001
Diabetes mellitus	39,883 (8%)	121,587 (13%)	0.165	38,470 (9%)	38,393 (9%)	0.001
Cardiovascular procedure(s)	105,663 (22%)	274,789 (30%)	0.191	98,380 (23%)	98,256 (23%)	0.001
Systemic connective tissue disorders	6939 (1%)	13,205 (1%)	0.002	6155 (1%)	6193 (1%)	0.001
American Indian or Alaska Native	664 (0%)	1834 (0%)	0.016	659 (0%)	646 (0%)	0.001
Dizziness	26,992 (6%)	69,210 (8%)	0.082	25,146 (6%)	25,052 (6%)	0.001
Aortic aneurysm and/or dissection	1495 (0%)	2906 (0%)	0.002	1326 (0%)	1293 (0%)	0.001
Hispanic or Latino	25,101 (5%)	89,488 (10%)	0.177	25,067 (6%)	24,921 (6%)	0.001
Other congenital syndromes	276 (0%)	577 (0%)	0.003	238 (0%)	253 (0%)	0.001
Nicotine dependence	31,043 (6%)	145,717 (16%)	0.307	30,945 (7%)	30,763 (7%)	0.002
Arterial fibromuscular dysplasia	67 (0%)	165 (0%)	0.003	57 (0%)	49 (0%)	0.002
Other coagulation defects	3245 (1%)	6917 (1%)	0.011	2843 (1%)	2902 (1%)	0.002
Adverse social determinants	7299 (1%)	30,353 (3%)	0.119	7042 (2%)	6946 (2%)	0.002
External causes of morbidity	31,861 (7%)	101,361 (11%)	0.160	30,492 (7%)	30,297 (7%)	0.002
Acute upper respiratory infection	129,742 (26%)	223,426 (24%)	0.050	111,524 (26%)	111,131 (26%)	0.002
Hyperlipemia	66,962 (14%)	140,506 (15%)	0.046	60,967 (14%)	61,295 (14%)	0.002
Visit: Inpatient	27,838 (6%)	142,828 (16%)	0.324	27,673 (6%)	27,909 (6%)	0.002
Black or African American	21,151 (4%)	206,025 (22%)	0.552	21,151 (5%)	21,361 (5%)	0.002
Hypertensive diseases	101,789 (21%)	262,614 (29%)	0.181	95,281 (22%)	95,697 (22%)	0.002
Family history of heart or circulatory disease	11,503 (2%)	25,195 (3%)	0.025	10,203 (2%)	10,049 (2%)	0.002
Diseases of arteries, arterioles and capillaries	15,167 (3%)	31,440 (3%)	0.018	13,628 (3%)	13,425 (3%)	0.003
Migraine	30,590 (6%)	67,320 (7%)	0.043	28,382 (7%)	28,087 (7%)	0.003
White	271,823 (55%)	396,477 (43%)	0.249	237,731 (55%)	238,385 (55%)	0.003
Native Hawaiian or Other Pacific Islander	406 (0%)	1513 (0%)	0.023	406 (0%)	366 (0%)	0.003
Autoimmune thyroiditis	6328 (1%)	7105 (1%)	0.051	5048 (1%)	4888 (1%)	0.003
Quinolones	38,750 (8%)	102,172 (11%)	0.109	37,017 (9%)	37,466 (9%)	0.004
Overweight and obesity	66,599 (14%)	165,287 (18%)	0.121	60,940 (14%)	61,550 (14%)	0.004
Antimigraine agents	17,093 (3%)	40,716 (4%)	0.048	16,421 (4%)	16,795 (4%)	0.005
Pregnancy	21,248 (4%)	101,371 (11%)	0.253	21,170 (5%)	21,614 (5%)	0.005
Homocystinuria	568 (0%)	577 (0%)	0.018	439 (0%)	371 (0%)	0.005
Systemic contraceptives	46,002 (9%)	86,922 (9%)	0.002	41,675 (10%)	42,755 (10%)	0.008
Visit: Emergency	105,441 (22%)	399,837 (44%)	0.483	104,468 (24%)	102,738 (24%)	0.009
Not Hispanic or Latino	329,292 (67%)	543,534 (59%)	0.168	284,984 (66%)	287,527 (67%)	0.012
Cardiovascular medication(s)	138,755 (28%)	376,059 (41%)	0.267	134,949 (31%)	138,793 (32%)	0.019

After age at index and sex, variables are sorted by increasing SMD. Abbreviations: spinal manipulative therapy (SMT); standardized mean difference (SMD); not applicable (NA)

### Primary and secondary outcomes

**Fig. 1:**
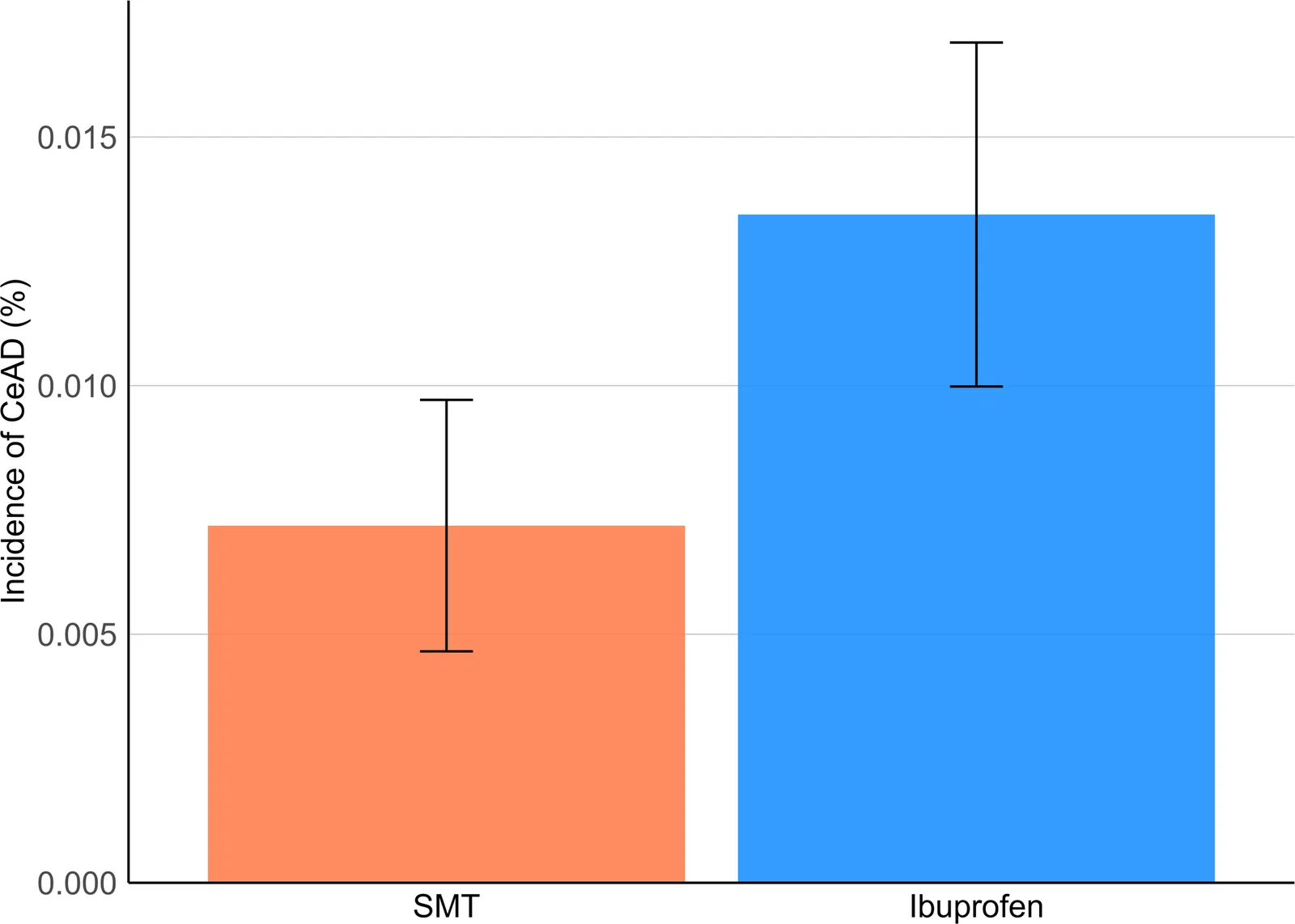
Total incidences of cervical artery dissection (CeAD) per cohort. The spinal manipulative therapy (SMT) cohort is represented in orange while the ibuprofen cohort is represented in blue. The error bars indicate 95% confidence intervals. Data represent findings after matching, over the 30-day follow-up window

**Fig. 2:**
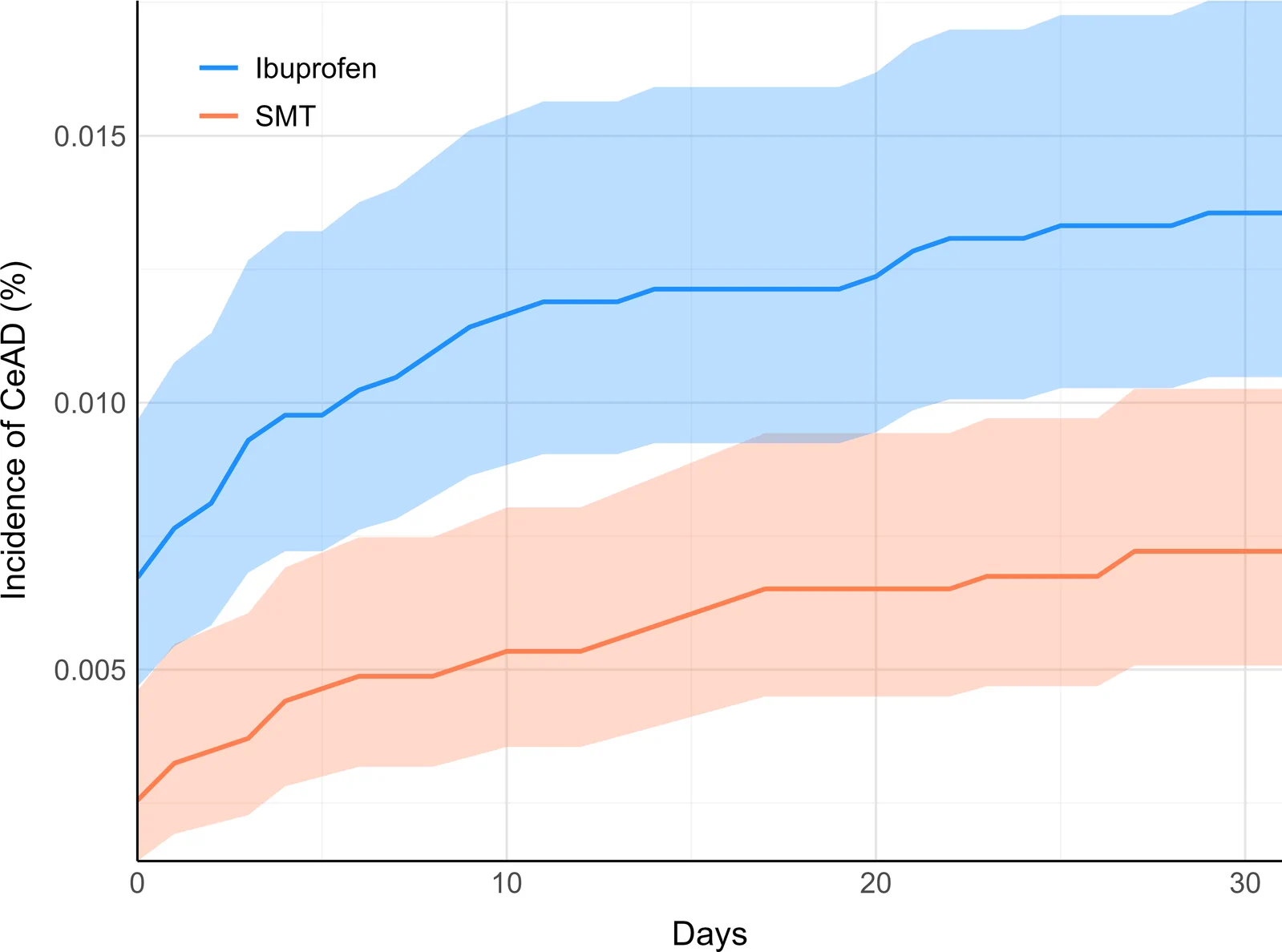
Cumulative incidences of cervical artery dissection (CeAD). The spinal manipulative therapy (SMT) cohort is represented in orange while the ibuprofen cohort is represented in blue. Shaded regions highlight 95% confidence intervals (CI). Data represent findings after matching, over the 30-day follow-up window. Both cohorts show similar curvilinear patterns with some events occurring on the index date (day 0), and maintain relatively consistent separation throughout follow-up with some overlap in 95% CI

**Fig. 3:**
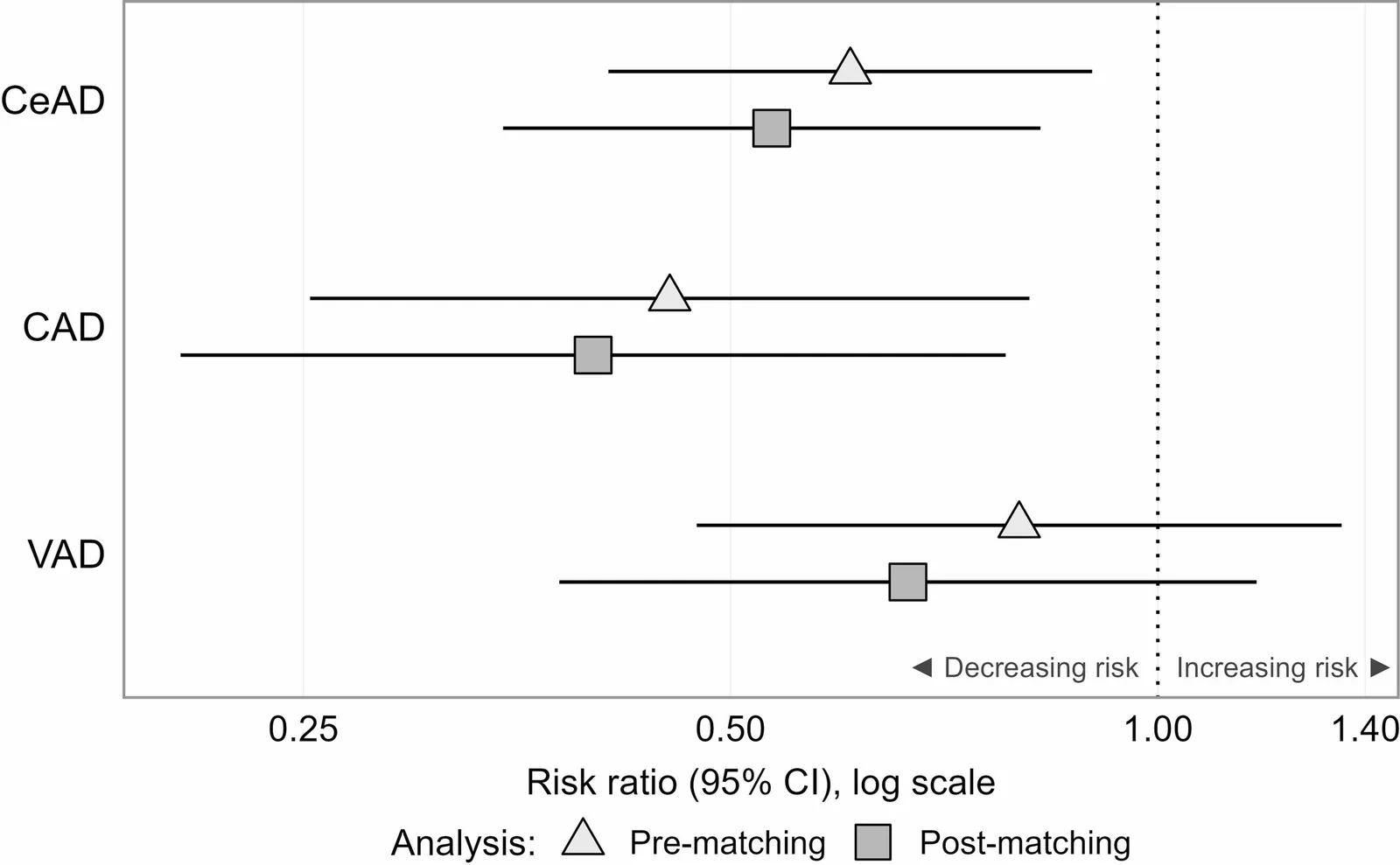
Risk ratio point estimates and 95% confidence intervals (CI; error bars) for key outcomes. Findings are shown before and after propensity score matching (pre-matching: triangles; post-matching: squares). Comparing spinal manipulative therapy recipients to ibuprofen recipients, values to the left of the null value of 1.0 (dotted line) suggest a potential reduction in risk of the outcome (◄), whereas values to the right suggest a potential increase in risk (►). CIs intersect or fall below the null, consistent with no statistically significant increase in risk. Abbreviations: cervical artery dissection (CeAD), carotid artery dissection (CAD), and vertebral artery dissection (VAD)

After matching, 31 SMT (0.007%) and 58 ibuprofen (0.013%) recipients had new CeAD during the 30-day follow-up. Comparing SMT to ibuprofen, CeAD risk was lower [95% CI] (RR = 0.53 [0.35, 0.83]; risk difference =-6.3 [-10.8, -2.0] events per 100,000 patients; *P* = 0.004). Carotid artery dissection risk was lower (RR = 0.40 [0.20, 0.78]; *P* = 0.005]), while risk of vertebral artery dissection was not significantly different (RR = 0.67 [0.38, 1.17]; *P* = 0.157]). RR estimates were relatively stable pre- and post-matching. Complete results are in Table 2. Total and cumulative incidences and RRs are shown in Figs. 1 and 2, and Fig. 3. CeAD subtype plots are shown in Figure S4 through S7.

**Table 2:** Outcome findings

	Before matching		After matching	
**Outcome and measure**	SMT	Ibuprofen	SMT	Ibuprofen
Total patients n	489,838	919,171	431,492	431,492
**Cervical artery dissection (CeAD)** ^*^				
CeAD n	33	102	31	58
CeAD % (95% CI)	0.007% (0.004%, 0.009%)	0.011% (0.009%, 0.013%)	0.007% (0.005%, 0.010%)	0.013% (0.010%, 0.017%)
CeAD n per 100,000 (95% CI)	6.7 (4.4, 9.0)	11.1 (8.9, 13.3)	7.2 (4.7, 9.7)	13.4 (10.0, 16.9)
Risk difference; n per 100,000 (95% CI; P-value)	-4.4 (-7.5, -1.0; *P* = 0.012)	reference	-6.3 (-10.8, -2.0; *P* = 0.004)	reference
Risk ratio (95% CI; P-value)^**a**^	0.61 (0.41, 0.90; *P* = 0.012)	reference	0.53 (0.35, 0.83; *P* = 0.004)	reference
**Carotid artery dissection (CAD)** ^**†**^				
CAD n	14	58	12	30
CAD % (95% CI)	0.003% (0.001%, 0.004%)	0.006% (0.005%, 0.008%)	0.003% (0.001%, 0.004%)	0.007% (0.004%, 0.009%)
CAD n per 100,000 (95% CI)	2.9 (1.4, 4.4)	6.3 (4.7, 7.9)	2.8 (1.2, 4.4)	7.0 (4.5, 9.4)
Risk difference; n per 100,000 (95% CI; P-value)	-3.5 (-5.7, -1.1; *P* = 0.006)	reference	-4.2 (-7.4, -1.3; *P* = 0.005)	reference
Risk ratio (95% CI; P-value)	0.45 (0.25, 0.81; *P* = 0.006)	reference	0.40 (0.20, 0.78; *P* = 0.005)	reference
**Vertebral artery dissection (VAD)** ^**†**^				
VAD n	20	47	20	30
VAD % (95% CI)	0.004% (0.002%, 0.006%)	0.005% (0.004%, 0.007%)	0.005% (0.003%, 0.007%)	0.007% (0.004%, 0.009%)
VAD n per 100,000 (95% CI)	4.1 (2.3, 5.9)	5.1 (3.7, 6.6)	4.6 (2.6, 6.7)	7.0 (4.5, 9.4)
Risk difference; n per 100,000 (95% CI; P-value)	-1.0 (-3.3, 1.5; *P* = 0.398)	reference	-2.3 (-5.7, 0.9; *P* = 0.157)	reference
Risk ratio (95% CI; P-value)	0.80 (0.47, 1.35; *P* = 0.398)	reference	0.67 (0.38, 1.17; *P* = 0.157)	reference

Abbreviations: spinal manipulative therapy (SMT), cervical artery dissection (CeAD), primary outcome (*), secondary outcome (†), 95% confidence interval (CI), carotid artery dissection (CAD), vertebral artery dissection (VAD)

### Follow-up care

After matching, SMT recipients had a mean (SD) 3.6 (3.0) SMT visits (median = 3) between the index date and 30-day follow-up window, translating to approximately 1,553,371 SMT visits. Ibuprofen recipients had a mean of 1.2 (0.7) ibuprofen prescriptions (median = 1). Comparing SMT to ibuprofen recipients, SMT recipients were slightly less likely to undergo physical therapy evaluation (2.2% vs. 3.2%; RR = 0.88 [95% CI: 0.86, 0.91]; Figure S8).

Some between-cohort crossover during follow-up after the index occurred. Specifically, 5.11% of the ibuprofen cohort received SMT between days 7 and 30, while 1.87% of the SMT cohort received ibuprofen during the 30-day window. Event rates for patients with crossover was similar to the original cohorts. SMT recipients receiving ibuprofen had a mean of 1.2 (0.7) ibuprofen prescriptions (median = 1), while ibuprofen recipients who received SMT had a mean (SD) 3.4 (2.8) SMT visits (median = 2).

### Time-to-event analysis

Time-to-event analyses yielded findings consistent with the RR estimates for the primary and secondary outcomes. Compared with the ibuprofen cohort, the SMT cohort had a lower hazard of CeAD (HR = 0.53 [95%CI: 0.34, 0.82]; *P* = 0.004) and carotid artery dissection (HR = 0.40 [0.20; 0.78]; *P* = 0.005), while the risk of vertebral artery dissection was not significantly different (HR = 0.66 [0.38; 1.17]; *P* = 0.151). There was no evidence that the proportional hazards assumption was violated for CeAD, carotid artery dissection, or vertebral artery dissection (*P* = 0.199, *P* = 0.894, and *P* = 0.084, respectively).

### Data quality

Post-matching, SMD values were optimal and propensity score densities overlapped (Figure S9 and S10). Unknown age or sex was 0% in both cohorts, while unknown ethnicity (30%; SMD = 0.005) and race (28%; SMD = 0.012) were nearly identical. Mean follow-up duration was slightly longer in the SMT versus ibuprofen cohort (SD) (29.7 [2.6] vs. 29.3 [4.2] days; SMD = 0.110). Complete follow-up was high (SMT: 98.5%; ibuprofen: 97.1%; Figure S11). Comparing the SMT to ibuprofen cohort, RRs for colonoscopy (RR = 1.07) and lower extremity MRI (RR = 0.74) were balanced, whereas appendectomy (RR = 0.24) and foreign body removal (RR = 0.30) were imbalanced (Figure S12).

## Discussion

Among adults with new neck pain or headache, we found no increased CeAD risk within 30 days following chiropractor-administered SMT compared to receiving ibuprofen in an outpatient setting. The 95% CI for the absolute risk difference was narrow (approximately  -11 to -2 CeAD cases per 100,000 patients) and excluded the a priori detectable difference of 0.03%. Our null hypothesis was that there would be no significant difference in risk between cohorts. We failed to reject that hypothesis. In clinical terms, these findings indicate no evidence of a meaningful increase in CeAD risk following SMT compared with ibuprofen. Secondary outcomes of carotid and vertebral artery dissections likewise supported these conclusions. Cumulative incidences were curvilinear across outcomes/cohorts, suggesting an increase in CeAD after care-seeking irrespective of treatment received. Additionally, the post hoc time-to-event analyses yielded HRs that were nearly identical to the primary RR estimates. There was no evidence that the proportional hazards assumption was violated, suggesting that the relative hazard between cohorts remained approximately constant throughout the 30-day follow-up window. Any apparent reduction in risk in SMT recipients was small and likely due to residual confounding, while meaningful increases in risk appear unlikely. Accordingly, these findings are conceptually more consistent with a non-inferiority framework than a superiority framework, as they provide no evidence of increased risk rather than evidence of superiority.

In our study, CeAD incidence was 7 to 13 per 100,000 over 30 days. Specifically, our cumulative incidence curve suggests that occurrence rises sharply in the first two weeks and then begins to plateau, but it is uncertain how incidence changes beyond 30 days. Our findings are consistent with the hypothesis that adults presenting with new neck pain or headache may have a transiently elevated baseline risk of CeAD irrespective of treatment. This interpretation is further supported by the lack of evidence linking ibuprofen to cerebrovascular events [47, 48]. Additional research is needed to clarify the magnitude and temporal association between neck pain and/or headache and CeAD across care settings.

Our findings are consistent with two large studies that implemented both case-crossover and case-control analyses among patients presenting with neck pain or headache and found no significant association between SMT and CeAD [74, 75]. Smaller studies reported positive associations, but did not require patients to have neck pain or headache, thereby potentially introducing confounding [11, 20]. Another case-control study found a positive SMT-CeAD association using hospitalized stroke patients as controls [14], which potentially inflated the observed association as these individuals are unlikely to receive SMT [22, 23, 76]. Accordingly, the inconsistency across previous studies’ results could be accounted for by differences in comparators and baseline symptoms.

Further research is needed to help clinicians distinguish CeAD from benign causes of neck pain and headache, such as migraine or mechanical neck pain [18]. This may include validation of prediction models incorporating CeAD signs and symptoms (e.g., acute/sudden onset, unusual or unfamiliar headache/neck pain, recent trauma or infection, and neurological signs) [77], and specificity of broader “red flags” of vascular headache [78], and “green flags” of benign primary headache (e.g., childhood onset, menstrual association, headache-free days, family history, stable pattern) [79]. Several CeAD risk factors including pregnancy, systemic lupus erythematosus, oral contraceptive use, and upper respiratory infection have been examined by few studies, or have a low to very low certainty of evidence [5, 80, 81], warranting further investigation.

Additional studies are warranted to assess whether our findings generalize to subgroups with specific CeAD risk factors who were underrepresented in the present study, such as migraine, connective tissue disorders, or those who are pregnant [5, 80]. Replication using alternate active comparators (e.g., physical therapy, other medications) or models (e.g., multivariable Cox proportional hazards or propensity weighting) could also be informative. Specifically, future studies might examine whether findings generalize to osteopathic manipulative treatment or physical therapist-administered manual therapy, which would require consideration of procedure code specificity and differences in patient populations across provider types. Finally, research should examine whether SMT is associated with stroke, a related outcome that could not be reliably assessed herein. While a meta-analysis found no strong evidence of a causal association between SMT and stroke, this was based on very low quality evidence, being hindered by indirectness and risk of bias [82].

### Strengths and limitations

The present study is strengthened by adherence to a registered protocol, with transparent reporting of all analytic additions beyond the pre-specified methods. Additional strengths include the active comparator design and multidisciplinary team (orthopedics, physical therapy, neurosurgery, cardiology, biostatistics, and chiropractic). The SMT cohort demographics (mean age in the 40s and female predominance) align with those reported in prior studies of chiropractic patients [4].

Data quality assessments were largely reassuring. Over half of covariates were balanced at baseline, with the remainder balanced post-matching, supporting a similar baseline CeAD propensity given our eligibility criteria alone. RRs and risk differences changed minimally from pre- to post-matching, supporting the stability of outcomes and further suggesting eligibility criteria achieved partial cohort balance. Loss to follow-up was minimal (< 3%), though slightly lower in the SMT cohort. Two of four negative control outcomes (colonoscopy and lower extremity MRI) were balanced, supporting similar diagnostic testing rates. However, SMT recipients had lower risks of appendectomy and foreign body removal, possibly due to residual unmeasured between-cohort differences. These outcomes should not be over-interpreted given their exploratory nature, without CeAD association, and a potential positive association between non-steroidal anti-inflammatory drugs and appendicitis risk [83]. Overall, data quality supports our findings, although unmeasured confounding remains possible.

Follow-up care metrics supported the plausibility of our findings and cohort comparability. In the SMT cohort, the mean of 3.6 chiropractic SMT visits over the 30-day follow-up aligns with utilization patterns documented in large national cohorts of chiropractic patients with neck or low back pain [84]. This frequency is also broadly consistent with chiropractic guideline recommendations of one to two visits per week for headache [51]. Furthermore, while only a minority of patients received a physical therapy evaluation, these proportions were similar across both cohorts and comparable to previously described utilization rates from a national sample [85]. Finally, ibuprofen prescription rates suggested clinical plausibility in both cohorts, indicating a right skew (mean > median) where most patients received a single prescription while a subset had additional orders [86].

As an observational study, these findings cannot fully exclude an SMT-CeAD association. The reduction in risk was statistically significant for carotid artery dissection but not vertebral artery dissection, which may reflect statistical imprecision given small event counts, unmeasured confounding, or could suggest differential associations by dissection subtype, warranting further investigation.

Selection of index treatments in outpatient settings, combined with several exclusionary criteria, aimed to identify relatively homogenous encounters where acute cerebrovascular events such as CeAD were unlikely to be suspected across both cohorts. However, this inference relies on clinical reasoning rather than direct free text natural language processing or chart review.

Propensity score matching excluded 12% of patients in the SMT cohort and 53% in the larger ibuprofen cohort, including a small number of CeAD events. Effect estimates were similar before and after matching, suggesting that trimming had limited influence on the observed association. Regardless, matching may underrepresent patients with uncommon risk factors compared with weighting approaches that retain all eligible observations. Future studies using inverse probability weighting should further evaluate the SMT-CeAD association and the robustness of these findings.

Unmeasured confounding likely explains the small apparent reduction in CeAD risk associated with spinal SMT. Several confounders exist that, individually or collectively, could meet the residual confounding threshold required to bring the upper confidence interval to include the null (E-value estimate: 3.18; limit closest to the null: 1.70). Chiropractors may selectively avoid cervical SMT in patients perceived to be at higher cerebrovascular risk based on clinical findings or family histories that are not well captured by diagnosis codes [16, 24]. A recent review of CeAD risk factors highlighted two variables with moderate certainty of evidence: migraine, which was balanced in our model, and genetic predispositions (specifically methylenetetrahydrofolate reductase polymorphisms), which were not reliably coded or available for propensity methods [5, 31]. Additionally, while we matched on fibromuscular dysplasia, this condition is often underdiagnosed [87]; and no code exists for a family history of CeAD. The TriNetX platform requires a uniform covariate assessment window and enforces date obfuscation, preventing matching on exact infection timings or seasonal variation [5]. Another unmeasured variable includes minor undiagnosed cervical trauma [59, 60], although more substantial traumas, ostensibly captured by “External causes of morbidity,” were optimally balanced. Higher education has been linked to increased CeAD diagnosis [88], which could reduce observed SMT risk if such patients were more aware of CeAD symptoms and avoided SMT. Differences in workup or referral strategies could influence detection, leading to undiagnosed CeAD (false negatives), although this seems unlikely given the similarity of negative control outcomes reflecting diagnostic testing (e.g., colonoscopy, lower extremity MRI). Given limited data granularity, we were unable to match on exact symptom duration or acute/sudden onset [77], though both cohorts were defined using new care-seeking episodes. We also lacked index-visit neurological examination findings and patient-reported measures of symptom severity, disability, or headache burden [77]. However, existing evidence does not support pain severity as a predictive factor for CeAD risk [5], with one study finding no difference in the proportion of moderate-to-severe pain between CeAD cases and controls [77]. Media coverage linking SMT and CeAD [89, 90] may have influenced health-seeking patterns [89, 90]. Finally, while SMT recipients had lower risks of appendectomy and foreign body removal suggesting potential differences in acute care seeking, these rare outcomes may reflect chance or visit clustering rather than true behavioral differences.

Examining dose-response relationships was not feasible using aggregated data. While a 30-day window could introduce confounding from other exposures [91], cumulative incidence plots suggested similar relationships at earlier time points. Due to limited data granularity, we were unable to determine specific biomechanics of SMT (e.g., force/amplitude, patient positioning, manual versus instrument-assisted), or stratify by technique, in which CeAD risk could vary. We were unable to incorporate a screening measure of blunt cerebrovascular injury or ascertain minor undiagnosed traumas aside from SMT.

Our 30-day washout for neck pain and headache diagnoses aimed to capture new care-seeking episodes. However, administrative data cannot guarantee the complete absence of antecedent symptoms, and some patients may have had subclinical or unreported neck pain prior to the index visit. The threshold was selected based on evidence from neck pain trajectory research [54] and is intentionally symmetric with our outcome ascertainment window, though a longer washout period may warrant exploration in future studies with larger datasets.

In our SMT cohort, a dual-code requirement was used to ostensibly improve cervical SMT exposure specificity relative to prior approaches relying on provider type or generic SMT codes alone [12–14, 26–28, 74, 75]. However, the accuracy of our cervical SMT exposure definition cannot be validated without chart review. Misclassification is possible in at least two ways. First, a chiropractor may have documented a cervical segmental dysfunction code (i.e., M99.01) along with the SMT, yet performed SMT exclusively on non-cervical spinal regions, without actually rendering cervical SMT. This practice would be inconsistent with typical coding practices [92], and could result in inclusion of patients who did not receive cervical SMT. Second, SMT CPT codes do not exclude the possibility that non-manipulative procedures were miscoded as SMT, potentially introducing heterogeneity in the exposure. Third, requiring a selective cervical SMT definition, having both M99.01 and at least one neck pain, cervical disorder, or headache code (e.g., cervicalgia, radiculopathy, tension-type headache), may have restricted the SMT cohort sample size, as chiropractors may not have used the M99.01 code despite rendering cervical SMT. The frequency of these scenarios in real-world billing data is unknown. Finally, co-occurrence of non-cervical SMT among otherwise eligible patients is unlikely to influence CeAD risk given the absence of a proposed mechanism linking non-cervical SMT to CeAD.

Similarly, the diagnostic accuracy of the ICD-10 code combinations used to identify eligible patients with neck pain or headache was not validated against medical record review, nor was this possible given the aggregate nature of the data. Some related diagnostic misclassification is therefore possible.

Our dataset-driven utilization rates of ibuprofen may under-represent actual use due to unrecorded over-the-counter use. Within the TriNetX platform, medication data primarily represent prescription orders. While supported by linkage to pharmacy claims [33, 34], the dataset does not confirm if ibuprofen was ultimately dispensed or consumed. Accordingly, our design did not restrict inclusion to ibuprofen-naïve individuals, which would be impractical given the common use of this medication even beyond neck pain or headache. Further, our eligibility criteria did not aim to include patients who exclusively self-medicated with over-the-counter ibuprofen at home. While missing these latter patients likely reduced our ibuprofen cohort’s sample size, and/or led to contamination between SMT and actual ibuprofen use, the ibuprofen cohort remained adequately powered. Because there is no known link between ibuprofen and CeAD [47, 48], ibuprofen was not evaluated as a risk exposure. Rather, the prescription served as an active comparator, behaving as a proxy for a clinical encounter wherein a patient sought evaluation for neck pain or headache and was managed with simple, first-line care. Therefore, variations in medication adherence, use, or temporal delays in filling the prescription are unlikely to bias our findings, as the primary exposure of interest was the care-seeking event itself, represented by receipt of cervical SMT versus first-line medical care as captured by a simultaneous ibuprofen prescription and outpatient visit.

Treatment crossover can attenuate the magnitude of observed associations [93], however, in this case the percentage of each cohort who crossed over was minimal (5.11% and 1.87%) [94], likely mitigating effects of this bias.

## Conclusions

This retrospective cohort study of 862,984 adults seeking care for a new episode of neck pain or headache found no evidence of an increased risk of CeAD within 30 days following chiropractor-administered cervical SMT compared with ibuprofen prescription. Observed CeAD incidences and time-to-event patterns tentatively suggest an elevated incidence among these patients irrespective of care. Despite robust data quality markers, residual confounding cannot be excluded. Clinicians, including chiropractors, should maintain vigilance for CeAD symptoms among these individuals. Further research is warranted on higher-risk subgroups (e.g., migraine), and stroke.

## Supplementary Information

Below is the link to the electronic supplementary material.

Supplementary Material 1

Supplementary Material 2

## Data Availability

De-identified, aggregated data obtained directly from TriNetX for baseline characteristics, propensity score densities, and for our primary outcome of CeAD, total and cumulative incidences, are available in a public figshare repository (https://doi.org/10.6084/m9.figshare.30338149) [95].
