# Supplementary material for: Association between spinal manipulative therapy and cervical artery dissection in patients with neck pain or headache: a retrospective cohort study
Source: Chiropr Man Therap. 2026 Sep 14;34:53. doi: 10.1186/s12998-026-00678-4 (PMC13576314; doi:10.1186/s12998-026-00678-4)
Supplement: Supplementary file 2 [file 12998_2026_678_MOESM2_ESM.docx]

Supplemental File for “Association between spinal manipulative therapy and cervical artery dissection in patients with neck pain or headache: a retrospective cohort study”

Robert J. Trager, Anthony N. Baumann, Collin M. Labak, Kelsey L. Lewis, Jaime Abraham Perez, Christine M. Goertz, Andres Schuster

Contents

[Figure S1: Study design elements	2](#_Toc236836419)

[Table S1: Neck pain and headache definition	3](#_Toc236836420)

[Table S2: Exclusion criteria	4](#_Toc236836421)

[Table S3: Propensity-matched variables	5](#_Toc236836422)

[Figure S2: Directed acyclic graph	6](#_Toc236836423)

[Table S4: Secondary outcomes	6](#_Toc236836424)

[Figure S3: Patient selection flowchart	7](#_Toc236836425)

[Figure S4: Total incidences of carotid artery dissection	8](#_Toc236836426)

[Figure S5: Total incidences of vertebral artery dissection	8](#_Toc236836427)

[Figure S6: Cumulative incidences of carotid artery dissection	9](#_Toc236836428)

[Figure S7: Cumulative incidences of vertebral artery dissection	9](#_Toc236836429)

[Figure S8: Total incidences of physical therapy evaluation	10](#_Toc236836430)

[Figure S9: Covariate balance plot	11](#_Toc236836431)

[Figure S10: Propensity score densities	12](#_Toc236836432)

[Figure S11: Loss to follow-up	12](#_Toc236836433)

[Figure S12: Negative control outcomes	13](#_Toc236836434)

[Table S5: E-values	13](#_Toc236836435)

[References	13](#_Toc236836436)

# Figure S1: Study design elements

The vertical arrow indicates the index date (day 0), when spinal manipulative therapy (SMT) or ibuprofen are received and eligibility criteria are met. Boxes and text descriptions show the eligibility criteria which were assessed during individual time windows ([#, #]) in days relative to the index date. Previous manual therapies and SMT exclusion extends to day 0 for the ibuprofen cohort (*). Other abbreviations: Cervical artery dissection (CeAD). Figure created using a Creative Commons template from Wang et al [1] (doi: 10.2147/CLEP.S358583).

# ***Table S1: Neck pain and headache definition***

Abbreviations: International Classification of Diseases, 10th Revision (ICD-10)

| **ICD-10 Codes** | **Definition** |
| --- | --- |
| **Neck pain** | |
| M50.1 | Cervical disc disorder with radiculopathy |
| M54.2 | Cervicalgia |
| M53.1 | Cervicobrachial syndrome |
| M53.82 | Other specified dorsopathies, cervical region |
| M54.12 | Cervical radiculopathy |
| **Headache** | |
| R51 | Headache |
| G43 | Migraine |
| G44.2 | Tension-type headache |
| G44.86 | Cervicogenic headache |
| M54.81 | Occipital neuralgia |

# Table S2: Exclusion criteria

Abbreviations: any preceding duration of time available in the patient’s data (-∞),Current Procedural Terminology (CPT); International Classification of Diseases, 10th Edition (ICD-10); ICD-10 Procedure Coding System (ICD-10-PCS); Systematized Nomenclature of Medicine (SNOMED); normalized names for clinical drugs (RxNorm)

| **Codes** | **Definition** | **Days** |
| --- | --- | --- |
| **Exclusions for both cohorts** | | |
| I72.6 (ICD-10) | Aneurysm of vertebral artery | -∞ to -1 |
| I77.71 (ICD-10) | Dissection of carotid artery | -∞ to -1 |
| I77.74 (ICD-10) | Dissection of vertebral artery | -∞ to -1 |
| I77.75 (ICD-10) | Dissection of other precerebral arteries | -∞ to -1 |
| G45 (ICD-10) | Transient cerebral ischemic attacks and related syndromes | -∞ to -1 |
| G46 (ICD-10) | Vascular diseases of brain in cerebrovascular diseases | -∞ to -1 |
| I60-I69 (ICD-10) | Cerebrovascular diseases (I60 - Nontraumatic subarachnoid hemorrhage; I61- Nontraumatic intracerebral hemorrhage; I62- Other and unspecified nontraumatic intracranial hemorrhage; I63 - Cerebral infarction; I65 - Occlusion and stenosis of precerebral arteries, not resulting in cerebral infarction; I66 - Occlusion and stenosis of cerebral arteries, not resulting in cerebral infarction; I67 - Other cerebrovascular diseases; I68 - Cerebrovascular disorders in diseases classified elsewhere; I69 - Sequelae of cerebrovascular disease) | -∞ to -1 |
| I97.81, I97.82 (ICD-10) | Intraoperative or postoperative cerebral infarction | -∞ to -1 |
| S15.0, S15.1 (ICD-10) | Injury of carotid artery of neck or vertebral artery | -∞ to -1 |
| A17, A18.01, G00-G09, M46.2, M46.3, M46.4, M46.5 (ICD-10) | Spinal infection: Tuberculosis of nervous system or spine; inflammatory diseases of the central nervous system (e.g., abscess); osteomyelitis of vertebra, infection of intervertebral disc (pyogenic), discitis, or other infective spondylopathies | -∞ to -1 |
| C41.2, C70, C72 (ICD-10) | Malignancy: of vertebral column, meninges, or spinal cord | -∞ to -1 |
| DWY1 (ICD-10-PCS) | Radiation therapy to head and neck | -∞ to -1 |
| M48.42; M48.52, M48.53; S12 | Cervical spinal fracture: Fatigue fracture of vertebra, cervical region; collapsed vertebra, cervical region or cervicothoracic region; fracture of cervical vertebra and other parts of neck | -∞ to -1 |
| R29.71, R29.72, R29.73, R29.74 (ICD-10) | National Institutes of Health Stroke Scale score 10-19; 20-29; 30-39; or 40-42 | -∞ to -1 |
| 8410, 259280 (RxNorm); J2997, J3101 (HCPCS); Z92.82 (ICD-10) | Recombinant tissue plasminogen activator (rtPA): Alteplase, tenectplase drug and injection codes; status-post administration of tpa (rtPA) | -∞ to -1 |
| 80051000119107 (SNOMED) | Stroke care management | -∞ to -1 |
| Z86.73 (ICD-10) | Personal history of transient ischemic attack (TIA), and cerebral infarction without residual deficits | -∞ to 0 |
| I69 (ICD-10) | Sequelae of cerebrovascular diseases (i.e., late effects) | -∞ to 0 |
| **Procedures and visits** | | |
| 1022228; 1022277 (CPT) | Transcatheter placement of stent: cervical carotid artery; extracranial vertebral artery | -∞ to -1 |
| 35301 (CPT) | Thrombarterectomy, including patch graft, if performed; carotid, vertebral, subclavian, by neck incision | -∞ to -1 |
| 03QJ, 03QH, 03QN, 03QM, 03QL, 03QK, 03QQ, 03QP (ICD-10-PCS) | Carotid or vertebral artery repair | -∞ to -1 |
| 1009028, 1014226, 38720, 38724, 1009060, 1006697  (CPT) | Anterior neck surgery: Thyroid excision procedures (e.g., lobectomy, thyroidectomy, biopsy), parathyroidectomy or exploration of parathyroid(s), cervical lymphadenectomy (complete or modified radical neck dissection), excision of carotid body tumor, insertion of central venous access device | -14 to -1 |
| 1013711 (CPT) | Emergency department services | -14 to -1 |
| 1013699 (CPT) | Inpatient or observation consultations | -14 to -1 |
| 1013729 (CPT) | Critical care services | -14 to -1 |
| 1004038 (CPT) | Surgical procedures on the spine (vertebral column) | -14 to -1 |
| 1009340 (CPT) | Surgical procedures on the spine and spinal cord | -14 to -1 |
| 1013558 (CPT) | Osteopathic manipulative treatment procedures | -30 to 0 |
| **Prior spinal manipulative therapy / ibuprofen cohort only** | | |
| 97140 (CPT) | Manual therapy techniques (e.g., mobilization/manipulation, manual traction) | -30 to 7 |
| 98940, 98941, 98942 (CPT) | Chiropractic manipulative treatment, spinal | -30 to 7 |

# Table S3: Propensity-matched variables

Abbreviations: International Classification of Diseases, 10th Revision (ICD-10), Veterans Affairs National Drug File (VA); positive association (**⬆**), negative association (**⬇**), variable association (**⬆⬇**).

| **Variable** | **Description** | **Association with CeAD** | **Reference(s)** |  |
| --- | --- | --- | --- | --- |
| **Demographics** | Age at index, current age, sex, race/ethnicity | **⬆⬇** | [2] |  |
| **Comorbidities (ICD-10)** | | | | |
| J00-J06 | Acute upper respiratory infections | **⬆** | [3,4] |  |
| Z55-Z65 | Adverse socioeconomic and psychosocial circumstances (i.e., social determinants of health) [5,6] | **⬇** | [3,7] |  |
| I71 | Aortic aneurysm and dissection | **⬆** | [8] |  |
| I77.3 | Arterial fibromuscular dysplasia | **⬆** | [9] |  |
| E06.3 | Autoimmune thyroiditis | **⬆** | [10,11] |  |
| F17 | Nicotine dependence | **⬆** | [12] |  |
| E08-E13 | Diabetes mellitus | **⬇** | [12–14] |  |
| R42 | Dizziness | **⬆** | [2] |  |
| I70-I79 | Diseases of arteries, arterioles, and capillaries (includes aneurysms, arterial embolism, arteritis, and arterial fibromuscular dysplasia) | **⬆** | [8,9] |  |
| Q79.6 | Ehlers-Danlos syndromes | **⬆** | [9] |  |
| Z82.4 | Family history of ischemic heart disease and other diseases of the circulatory system | **⬆** | [13] |  |
| E72.11 | Hyperhomocysteinemia | **⬆** | [15] |  |
| E78.5 | Hyperlipidemia, unspecified | **⬇** | [12,13,16] |  |
| I10-I1A | Hypertensive diseases | **⬆** | [13] |  |
| F10-F19 | Mental and behavioral disorders due to psychoactive substance use | **⬆** | [17,18] |  |
| G43 | Migraine | **⬆** | [13] |  |
| D68 | Other coagulation defects (includes antiphospholipid syndrome, lupus anticoagulant syndrome) | **⬆** | [10,19] |  |
| Q87 | Other specified congenital syndromes (includes Marfan syndrome, Loeys Dietz syndrome, Alport syndrome, arterial tortuosity syndrome) | **⬆** | [9,20] |  |
| E66 | Overweight and obesity | **⬇** | [13] |  |
| O00-O9A | Pregnancy, childbirth and the puerperium | **⬆** | [21] |  |
| R55 | Syncope | **⬆** | [2] |  |
| M30-M36 | Systemic connective tissue disorders (includes lupus, Sjögren’s syndrome, hypermobility syndrome) | **⬆** | [22,23] |  |
| V00-Y99 | External causes of morbidity (e.g., trauma) | **⬆** | [9] |  |
| **Visits and procedures** | | | |  |
| EMER* | Emergency visit | NA | NA |  |
| IMP* | Inpatient visit | NA | NA |  |
| 1012974 | Cardiovascular procedures (e.g., echocardiography, electrocardiogram) | NA | NA |  |
| **Treatments** | | | | |
| CN105 (VA) | Antimigraine agents (includes triptans) | **⬆** | [24] |  |
| CV000 (VA) | Cardiovascular medications (e.g., beta blockers, calcium channel blockers, anti-arrhythmics, antilipemic agents, antihypertensives, diuretics, angiotensin-converting enzyme inhibitors inhibitors, angiotension II inhibitors) | **⬇** | [13,16] |  |
| HS200 (VA) | Contraceptives, systemic | **⬆** | [25,26] |  |
| AM400 (VA) | Quinolones (includes fluoroquinolones) | **⬆** | [27] |  |

# Figure S2: Directed acyclic graph

*This graph maps potential causal relationships between exposures of spinal manipulative therapy (SMT) or ibuprofen (square □), other confounders (circles ○), and cervical artery dissection (CeAD, triangle △). Circles represent confounders that are controlled for via matching (i.e., medications, comorbidities, migraine, socioeconomic status [SES], age, and sex). Some confounders also influence selection bias due to having an association with likelihood of using SMT* [28,29]*. Arrows indicate causal directions. Graph created using dagitty and ggdag in R* [30,31]*.*

# Table S4: Secondary outcomes

Abbreviations: International Classification of Diseases, 10th Revision (ICD-10), ICD-10 Procedure Coding System (ICD-10-PCS); Current Procedural Terminology (CPT); Systematized Nomenclature of Medicine (SNOMED); normalized names for clinical drugs (RxNorm)

| **Variable** | **Description** |
| --- | --- |
| **Cervical artery dissection subtypes** | |
| I77.71 (ICD-10) | Dissection of carotid artery |
| I77.74 (ICD-10) | Dissection of vertebral artery |
| **Negative control outcomes** | |
| 1022231 (CPT) | Colonoscopy, flexible |
| 44970, 1014622 (CPT); 0DTJ (ICD-10-PCS) | Appendectomy |
| 10120 (CPT) | Incision and removal of foreign body, subcutaneous tissues; simple |
| 73721 (CPT) | Magnetic resonance (eg, proton) imaging, any joint of lower extremity; without contrast material |
| **Follow-up care** | |
| 98940, 98941, 98942 (CPT) | Chiropractic manipulative treatment (CMT); spinal, 1-2 regions, 3-4 regions, or 5 regions |
| 5640 (RxNorm) | Ibuprofen prescription |
| 1029677 (CPT) | Physical therapy evaluations |

# Figure S3: Patient selection flowchart

This diagram illustrates the cohort size derivations from the entire TriNetX Linked sample, and the impact of specific exclusionary criteria on cohort size. The spinal manipulative therapy (SMT) cohort is shown in orange, while the Ibuprofen cohort is shown in blue. Exclusions are indicated with an asterisk (*) on the left. Trimming during propensity score matching led to a 12% reduction in the SMT cohort size and 53% reduction in the ibuprofen cohort size.

# Figure S4: Total incidences of carotid artery dissection

The spinal manipulative therapy (SMT) cohort is represented in orange while the ibuprofen cohort is represented in blue. The error bars indicate 95% confidence intervals. Data represent findings after matching, over the 30-day follow-up window.

# Figure S5: Total incidences of vertebral artery dissection

The spinal manipulative therapy (SMT) cohort is represented in orange while the ibuprofen cohort is represented in blue. The error bars indicate 95% confidence intervals. Data represent findings after matching, over the 30-day follow-up window.

# Figure S6: Cumulative incidences of carotid artery dissection

The spinal manipulative therapy (SMT) cohort is represented in orange while the ibuprofen cohort is represented in blue. Shaded regions highlight 95% confidence intervals. Data represent findings after matching, over the 30-day follow-up window.

# Figure S7: Cumulative incidences of vertebral artery dissection

The spinal manipulative therapy (SMT) cohort is represented in orange while the ibuprofen cohort is represented in blue. Shaded regions highlight 95% confidence intervals. Data represent findings after matching, over the 30-day follow-up window.

# Figure S8: Total incidences of physical therapy evaluation

The spinal manipulative therapy (SMT) cohort is represented in orange while the ibuprofen cohort is represented in blue. Shaded regions highlight 95% confidence intervals. Data represent findings after matching, over the 30-day follow-up window.

# Figure S9: Covariate balance plot

This plot shows standardized mean differences (SMDs) before and after matching. The vertical dashed line coinciding at SMD=0.1 indicates the threshold for optimal balance [32,33]. Before-matching SMDs are shown as triangles, while SMDs after matching are shown as squares. Plot created using R and R studio (version 4.2.2, Vienna, AT [34]) and ggplot2 [35].

# Figure S10: Propensity score densities

Density scores before matching (A) and after matching (B). Orange bars represent the spinal manipulative therapy (SMT) cohort while blue bars represent the ibuprofen cohort. Following matching, densities are similar, indicating adequate balance of covariates.

# Figure S11: Loss to follow-up

Graph A *illustrates the proportion of patients remaining per cohort per over time, indicating the spinal manipulative therapy (SMT) cohort in orange and ibuprofen cohort in blue. Graph B shows the proportion of patients per cohort with data spanning at least the entire five-year follow-up (i.e., SMT: 97.1%; ibuprofen: 98.5%). Both cohorts had a median follow-up of 30 days with an interquartile range of zero. Plots generated using* R and R studio (version 4.2.2, Vienna, AT [34]) and ggplot2 [35].

# Figure S12: Negative control outcomes

Point risk ratio estimates and 95% confidence intervals (CI; error bars) are shown. The green area represents estimates from 0.73 to 1.38, indicating the predefined bounds for negative control outcome balance while the vertical dotted line indicates the null value of 1.0. Abbreviation: Magnetic resonance imaging (MRI).

# Table S5: E-values

| **Outcome** | **E-value** | **E-value for lower confidence interval** |
| --- | --- | --- |
| Cervical artery dissection | 3.18 | 1.70 |
| Carotid artery dissection | 4.44 | 1.88 |
| Vertebral artery dissection | 2.35 | 1.00 |

# References

1.	Wang SV, Schneeweiss S: A Framework for Visualizing Study Designs and Data Observability in Electronic Health Record Data. Clin Epidemiol. 2022, 14:601–8. 10.2147/CLEP.S358583

2.	Shu L, Strelecky L, de Havenon A, et al.: Incidence Trends and Risk of Recurrent Stroke of Cervical Artery Dissections in the United States Between 2005 and 2019. Neurology. 2025, 104:e213548. 10.1212/WNL.0000000000213548

3.	Grau AJ, Brandt T, Buggle F, et al.: Association of Cervical Artery Dissection With Recent Infection. Arch Neurol. 1999, 56:851–6. 10.1001/archneur.56.7.851

4.	Witsch J, Rutrick SB, Lansdale KN, et al.: Influenza-Like Illness as a Short-Term Risk Factor for Arterial Dissection. Stroke. 2023, 54:e66–8. 10.1161/STROKEAHA.122.042367

5.	Heidari E, Zalmai R, Richards K, Sakthisivabalan L, Brown C: Z-code documentation to identify social determinants of health among Medicaid beneficiaries. Res Soc Adm Pharm RSAP. 2023, 19:180–3. 10.1016/j.sapharm.2022.10.010

6.	Schulte A, Donovan AL, McAdam-Marx C: From access to equity: The devil is in ‘Z’ details. J Manag Care Spec Pharm. 2022, 28:685–7. 10.18553/jmcp.2022.28.6.685

7.	Kellert L, Grau A, Pezzini A, et al.: University education and cervical artery dissection. J Neurol. 2018, 265:1065–70. 10.1007/s00415-018-8798-7

8.	Witsch J, Mir SA, Parikh NS, et al.: Association Between Cervical Artery Dissection and Aortic Dissection. Circulation. 2021, 144:840–2. 10.1161/CIRCULATIONAHA.121.055274

9.	Debette S: Pathophysiology and risk factors of cervical artery dissection: what have we learnt from large hospital-based cohorts? Curr Opin Neurol. 2014, 27:20–8. 10.1097/WCO.0000000000000056

10.	Li H, Song P, Yang W, et al.: Association Between Autoimmune Diseases and Spontaneous Cervicocranial Arterial Dissection. Front Immunol. 2022, 12:820039. 10.3389/fimmu.2021.820039

11.	Pezzini A, Del Zotto E, Mazziotti G, et al.: Thyroid Autoimmunity and Spontaneous Cervical Artery Dissection. Stroke. 2006, 37:2375–7. 10.1161/01.STR.0000236500.15976.f3

12.	Abdelnour LH, Abdalla ME, Elhassan S, Kheirelseid EAH: Meta-analysis of cardiovascular risk profile of stroke secondary to spontaneous cervical artery dissection compared to ischemic stroke of other causes. Health Sci Rev. 2022, 5:100058. 10.1016/j.hsr.2022.100058

13.	Del Zotto E, Grassi M, Zedde M, et al.: Risk Profile of Patients with Spontaneous Cervical Artery Dissection. Ann Neurol. 2023, 94:585–95. 10.1002/ana.26717

14.	Engelter ST, Grond-Ginsbach C, Metso TM, et al.: Cervical artery dissection: trauma and other potential mechanical trigger events. Neurology. 2013, 80:1950–7. 10.1212/WNL.0b013e318293e2eb

15.	Gallai V, Caso V, Paciaroni M, Cardaioli G, Arning E, Bottiglieri T, Parnetti L: Mild Hyperhomocyst(e)inemia. Stroke. 2001, 32:714–8. 10.1161/01.STR.32.3.714

16.	Abdelnour LH, Abdalla ME, Elhassan S, Kheirelseid EAH: Diabetes, hypertension, smoking, and hyperlipidemia as risk factors for spontaneous cervical artery dissection: Meta-analysis of case-control studies. Curr J Neurol. 2022, 21:183–93. 10.18502/cjn.v21i3.11112

17.	Hori S, Hori E, Umemura K, et al.: Anatomical Variations of Vertebrobasilar Artery are Closely Related to the Occurrence of Vertebral Artery Dissection—An MR Angiography Study. J Stroke Cerebrovasc Dis. 2020, 29:104636. 10.1016/j.jstrokecerebrovasdis.2020.104636

18.	Smith WS, Johnston SC, Skalabrin EJ, Weaver M, Azari P, Albers GW, Gress DR: Spinal manipulative therapy is an independent risk factor for vertebral artery dissection. Neurology. 2003, 60:1424–8. 10.1212/01.WNL.0000063305.61050.E6

19.	Al-banaa K, Alshaikhli A, AL-Hareeri A, Abdelhalim M, Al-Hillan A, Joshi T: Arterial Dissection in Antiphospholipid Syndrome Patients: Two Case Reports and a Literature Review. Eur J Case Rep Intern Med. 2021, 8:002610. 10.12890/2021_002610

20.	Traenka C, Kloss M, Strom T, et al.: Rare genetic variants in patients with cervical artery dissection. Eur Stroke J. 2019, 4:355–62. 10.1177/2396987319861869

21.	Trager RJ, Daniels CJ, Scott ZE, Perez JA: Pregnancy and spontaneous cervical artery dissection: A propensity-matched retrospective cohort study. J Stroke Cerebrovasc Dis. 2023, 32:107384. 10.1016/j.jstrokecerebrovasdis.2023.107384

22.	Li H, Xu S, Xu B, Zhang Y, Yin J, Yang Y: Unraveling the Links between Chronic Inflammation, Autoimmunity, and Spontaneous Cervicocranial Arterial Dissection. J Clin Med. 2023, 12:5132. 10.3390/jcm12155132

23.	Trager RJ, Lynn BP, Baumann AN, Chu EC-P: Systemic lupus erythematosus is associated with an increased risk of cervical artery dissection. Sci Rep. 2025, 15:1194. 10.1038/s41598-025-85655-2

24.	Roberto G, Piccinni C, D’Alessandro R, Poluzzi E: Triptans and serious adverse vascular events: Data mining of the FDA Adverse Event Reporting System database. Cephalalgia. 2014, 34:5–13. 10.1177/0333102413499649

25.	D’Anglejan-Chatillon J, Ribeiro V, Mas J l., Youl B d., Bousser M g.: Migraine - A Risk Factor for Dissection of Cervical Arteries. Headache J Head Face Pain. 1989, 29:560–1. 10.1111/j.1526-4610.1989.hed2909560.x

26.	Trager RJ, Haering CP, Baumann AN, Wright DS: Association between combined oral contraceptive prescription and cervical artery dissection: A retrospective cohort study. Thromb Res. 2025, 247:109279. 10.1016/j.thromres.2025.109279

27.	Zotto ED, Pezzini A: Use of fluoroquinolones and the risk of spontaneous cervical artery dissection. Eur J Neurol. 2019, 26:1028–31. https://doi.org/10.1111/ene.13917

28.	Chevan J, Riddle DL: Factors associated with care seeking from physicians, physical therapists, or chiropractors by persons with spinal pain: a population-based study. J Orthop Sports Phys Ther. 2011, 41:467–76. 10.2519/jospt.2011.3637

29.	Adams J, Peng W, Cramer H, et al.: The Prevalence, Patterns, and Predictors of Chiropractic Use Among US Adults: Results From the 2012 National Health Interview Survey. Spine. 2017, 42:1810–6. 10.1097/BRS.0000000000002218

30.	Textor J, van der Zander B, Gilthorpe MS, Liskiewicz M, Ellison GT: Robust causal inference using directed acyclic graphs: the R package ‘dagitty’. Int J Epidemiol. 2016, 45:1887–94. 10.1093/ije/dyw341

31.	Barrett M: ggdag: Analyze and Create Elegant Directed Acyclic Graphs. 2024.

32.	Austin PC: Balance diagnostics for comparing the distribution of baseline covariates between treatment groups in propensity-score matched samples. Stat Med. 2009, 28:3083–107. 10.1002/sim.3697

33.	Stuart EA, Lee BK, Leacy FP: Prognostic score–based balance measures can be a useful diagnostic for propensity score methods in comparative effectiveness research. J Clin Epidemiol. 2013, 66:S84-S90.e1. 10.1016/j.jclinepi.2013.01.013

34.	R Core Team: R: A Language and Environment for Statistical Computing. R Foundation for Statistical Computing: Vienna, Austria; 2022.

35.	Wickham H: ggplot2: Elegant Graphics for Data Analysis. Springer-Verlag New York; 2016.
